# Transcriptome Analysis and Emerging Driver Identification of CD8+ T Cells in Patients with Vitiligo

**DOI:** 10.1155/2019/2503924

**Published:** 2019-11-26

**Authors:** Qiancheng Deng, Jingchao Wei, Puyu Zou, Yangfan Xiao, Zhuotong Zeng, Yaqian Shi, Yi Zhan, Huiming Zhang, Bingsi Tang, Qinghai Zeng, Rong Xiao

**Affiliations:** ^1^Department of Dermatology, Second Xiangya Hospital, Central South University, Hunan Key Laboratory of Medical Epigenetics, Changsha, China; ^2^Department of Urology, Third Xiangya Hospital of Central South University, Changsha, China; ^3^Department of Anesthesiology, Second Xiangya Hospital, Central South University, Changsha, China; ^4^Department of Dermatology, Third Xiangya Hospital of Central South University, Changsha, China

## Abstract

Activated CD8+ T cells play important roles in the pathogenesis of vitiligo. However, driving factors about the activation and migration of CD8+ T cells remain obscure. In this study, we aim to identify differentially expressed genes (DEGs) and uncover potential factors that drive the disease in melanocyte-specific CD8+ T cells in vitiligo. A total of 1147 DEGs were found through transcriptome sequencing in CD8+ T cells from lesional skin of vitiligo patients and normal controls. Based on KEGG pathway enrichment analysis and PPI, 16 upregulated and 23 downregulated genes were identified. Ultimately, 3 genes were figured out after RT-qPCR verification. The mRNA and protein expression levels of PIK3CB, HIF-1*α*, and F2RL1 were all elevated in CD8+ T cells from peripheral blood in vitiligo. HIF-1*α* and PIK3CB were significantly increased in lesional skin of vitiligo. Two CpG sites of the HIF-1*α* promoter were hypomethylated in vitiligo CD8+ T cells. In conclusion, HIF-1*α*, F2RL1, and PIK3CB may act as novel drivers for vitiligo, which are all closely associated with reactive oxygen species and possibly contribute to the activation and/or migration of melanocyte-specific CD8+ T cells in vitiligo. In addition, we uncovered a potential role for DNA hypomethylation of HIF-1*α* in CD8+ T cells of vitiligo.

## 1. Introduction

Vitiligo is an autoimmune skin disease characterized by depigmented skin due to the loss of melanocytes [1]. CD8+ T cells are cytotoxic T lymphocytes (CTLs) that kill target cells via secreting cytotoxic granules (perforin/granzyme B) or by Fas signaling [2, 3]. High levels of cytotoxic CD8+ T cells are detected in both the lesional skin and blood of vitiligo patients [4–6]. Importantly, activated CD8+ T cells have been confirmed to be melanocyte-specific T cells *in vivo* and *in vitro* for vitiligo patients [7–10]. Direct destruction of melanocytes has been demonstrated to be primarily correlated with CD8+ T cells [4, 5, 11]. The migration of circulating CD8+ T cells to sites of inflammation is an important part in the process of melanocyte destruction [12, 13], and most currently available evidence suggests that chemokines play an important role in regulating the homing of immune cells [14–16]. CXCL10 is a critical chemokine that triggers migration and probably modulates the cytotoxic functions of CD8+ T cells in vitiligo [14]. A mouse model study of vitiligo has indicated that transferred melanocyte-specific CD8+ T cells are activated and recruited to the skin via the expression of CXCR3 ligands [8]. This suggests an important recruitment role for the CXCL10/CXCR3 axis in melanocyte-specific CD8+ T cells [14]. However, it remains unknown exactly how these chemotactic CD8+ T cells are activated and proliferate in vitiligo. Therefore, we performed transcriptome analysis of CD8+ T cells from vitiligo lesional skin to identify differentially expressed genes (DEGs) and uncover potential driving factors for CD8+ T cells.

It is known that environmental factors, such as ultraviolet light and chemical exposure contribute to the production of damage-associated molecular pattern (DAMP) or pathogen-associated molecular pattern (PAMP) molecules. DAMPs or PAMPs trigger innate immunity by activating macrophages and dendritic cells, which ultimately result in adaptive immune responses and melanocyte destruction in vitiligo [5, 17]. In recent years, mounting evidence has demonstrated that epigenetic modifications play a critical role in autoimmune diseases triggered by environmental factors [18–20]. Epigenetics is defined as by heritable changes in gene expression that do not involve changes in the genomic DNA sequence [3, 21]. The three main epigenetic mechanisms are DNA methylation, histone modification, and microRNAs (miRNAs) [22]. DNA methylation is the most extensively studied epigenetic mechanism and is implicated in the silencing of gene expression [3]. Epigenetic mechanisms have been demonstrated to contribute to the development of autoimmune diseases, such as systemic lupus erythematosus [23, 24], rheumatoid arthritis [23, 25], systemic sclerosis [26, 27], multiple sclerosis [28], and type 1 diabetes [29, 30]. A study conducted by Zhao et al. reported that global DNA methylation levels are abnormal in peripheral blood mononuclear cells (PBMCs) of patients with vitiligo [31]. Based on these observations, DNA methylation-sensitive genes from DEGs may trigger the activation and proliferation of CD8+ T cells to initiate and promote melanocyte destruction in vitiligo via epigenetic mechanisms.

In the present study, we performed transcriptome sequencing of CD8+ T cells from the vitiligo lesional skin and normal controls and then screened for DEGs. We further investigated both the mRNA and protein expression levels of DEGs in CD8+ T cells from PBMCs and lesional skin in patients with vitiligo. We also validated the DNA methylation levels of the HIF-1*α* and F2RL1 promoter to explore the potential pathogenic mechanisms regarding CD8+ T cells in vitiligo. Taken together, our results provide novel insights into the pathogenesis of CD8+ T cells in vitiligo and the involvement of epigenetic mechanisms in promoting it.

## 2. Materials and Methods

### 2.1. Patients and Samples

Skin samples were obtained from 15 patients with vitiligo and 13 healthy controls in the Second Xiangya Hospital of Central South University. Skin samples of patients with vitiligo were obtained from lesional skin, and normal controls were collected from plastic surgery recipients. All patients enrolled in this study were diagnosed with nonsegmental vitiligo and were not treated with systemic therapy for at least 3 months or topical steroids for at least a month before obtaining the skin samples. Blood samples were collected from 19 patients with generalized vitiligo and 19 healthy controls. All patients and controls were matched for sex, age (±5 years), and ethnicity. All participants signed written informed consent forms. This study was reviewed and approved by the ethics committee of the Second Xiangya Hospital of Central South University.

### 2.2. CD8+ T Cell Isolation and Culture

Skin samples were placed into dispase II (10 mg/ml, Sigma, USA) for overnight incubation at 4°C to separate the dermis from the epidermis. Then, the separated dermis was digested with collagenase IV (1 mg/ml, Sigma, USA) for 1-2 h at 37°C. Single-cell suspensions were obtained from digested dermis suspensions through sterile cell strainers (pore size: 70 *μ*m, Falcon, USA). PBMCs were separated from the peripheral blood of patients with vitiligo and normal controls by density gradient centrifugation (Ficoll-Paque, GE Healthcare, Life Sciences, NJ, USA). CD8+ T cells were isolated from PBMCs and single-cell suspension by positive selection using CD8+ T MicroBeads, according to the manufacturer's protocol (Miltenyi Biotec, Germany). CD8+ T cell purity was confirmed by flow cytometry and was consistently higher than 95%. Then, CD8+ T cells isolated from single-cell suspensions were subjected to transcriptome sequencing. CD8+ T cells separated from PBMCs were cultured in RPMI 1640 medium (Gibco, USA) supplemented with 10% fetal bovine serum (HyClone, USA) and stimulated with plate-bound anti-CD3 (5 *μ*g/ml, Calbiochem, Germany) and anti-CD28 (2 *μ*g/ml, Calbiochem, Germany) for 72 h at 37°C and 5% CO_2_ or collected directly for subsequent experiments [32]. Normal CD8+ T cells from PBMCs were treated with 1 *μ*M 5-azacytidine (5-azaC, a DNA methyltransferase inhibitor, Sigma, USA) and cultured for 72 h [33, 34].

### 2.3. Transcriptome Sequencing

RNA was extracted from CD8+ T cells of skin samples using the TRIzol reagent (Invitrogen, USA). Transcriptome sequencing of CD8+ T cells from 5 vitiligo patients and 3 healthy controls was performed using the Illumina HiSeq X Ten platforms (Illumina, USA).

### 2.4. Data Analysis

Raw reads were filtered to remove low-quality reads and produce clean reads. Then, clean reads were mapped to a reference genome and genes using HISAT and Bowtie2, respectively. The expression of genes was calculated by FPKM (fragments per kilobase of transcript per million fragments mapped) with RSEM software. The DEGs were screened out by using the combined criteria of the absolute fold change ≥ 2 and *p* value < 0.01. Gene Ontology (GO) and Kyoto Encyclopedia of Genes and Genomes (KEGG) pathway enrichment analyses were performed to figure out the relevant functions and pathways in DEGs. *p* values in GO and KEGG analyses were corrected by a false discovery rate (FDR). A value of FDR ≤ 0.01 was defined as significant enrichment.

### 2.5. Quantitative Real-Time PCR (RT-qPCR)

Total RNA was extracted from CD8+ T cells using the TRIzol reagent. mRNA was reverse transcribed with 1 *μ*g of total RNA by using the FastQuant RT Kit with gDNase (Tiangen, China). RT-qPCR was performed with SuperReal PreMix Plus (Tiangen, China) using a LightCycler 96 thermocycler (Roche, Switzerland). The relative gene expression levels were normalized to the housekeeping gene *β*-actin and calculated by using the 2^−*ΔΔ*Ct^ method. Primers used in this study are shown in Supplemental [Supplementary-material supplementary-material-1].

### 2.6. Western Blotting

Total protein was extracted from the lysed CD8+ T cells. Protein concentration was determined using the Pierce™ Rapid Gold BCA Protein Assay Kit (Thermo Scientific, USA). Proteins were separated by 10% SDS-PAGE (Bio-Rad, USA) and transferred to polyvinylidene fluoride (PVDF) membranes (Millipore, USA). Membranes were blocked in 5% fat-free dried milk in phosphate-buffered saline containing 0.1% Tween-20 (PBST) solution and incubated with antibodies against HIF-1*α* (1 : 300; Proteintech, USA), PIK3CB (1 : 1000; Abcam, UK), and F2RL1 (1,1000; Abcam, UK). Band intensity was detected by using the ImageQuant™ LAS 4000 mini (GE Healthcare, USA).

### 2.7. Immunohistochemistry (IHC)

Skin tissue was fixed overnight in 4% paraformaldehyde (PFA) and embedded in paraffin. Paraffin sections were cut at a 4 *μ*m thickness. IHC was conducted by using the Opal™ 7-color manual kit (PerkinElmer, USA) according to the manufacturer's protocol. Antigen retrieval was performed in citrate buffer (pH 6.0) using a high-pressure method. Primary antibodies were incubated for 1 h in a humidified chamber at room temperature (RT), followed by incubation with a secondary HRP-conjugated antibody and Opal Fluorophore Working Solution (TSA, 1 : 100) for 10 min at RT. For Opal IHC, primary antibodies included antibodies against HIF-1*α* (1 : 100; Proteintech, USA), PIK3CB (1 : 100; Abcam, UK), and F2RL1 (1 : 100; Abcam, UK). Tissue slides were imaged using the Mantra Quantitative Pathology Imaging Systems (PerkinElmer, USA). Image analysis was performed with the InForm image analysis software (PerkinElmer, USA).

### 2.8. MethylTarget

Total DNA was extracted from CD8+ T cells using TIANamp Blood DNA Kit (Tiangen, China) according to the manufacturer's protocol. The DNA methylation status of the HIF-1*α* and F2RL1 promoter was analyzed using MethylTarget sequencing. Genomic DNA was converted with bisulfite treatment by using an EZ DNA Methylation-Gold Kit (Zymo, USA). PCR reactions were performed to amplify the targeted DNA sequences. Then, the products were sequenced by the Illumina Hiseq 2000. Bisulfite-treated reads and methylation calling were mapped by the BS-Seeker2 [35]. Primers used for PCR are shown in Supplemental [Supplementary-material supplementary-material-1]. DNA methylation levels of each CpG of DEGs were equal to the ratio of methylated cytosine to total cytosine.

### 2.9. Statistical Analysis

All data were processed with GraphPad Prism 6.0 (GraphPad Software, San Diego, CA). We measured data for normal distribution and similar variance between compared groups. Comparisons were analyzed using two-tailed unpaired Student's *t*-test between two groups and one-way analysis of variance (ANOVA) with Tukey post hoc tests for multiple comparisons. When the data were not normal distribution or showed unequal variances between two groups, we used the two-tailed Mann-Whitney *U* test for statistical analysis. The results are presented as means ± SEM. *p* < 0.05 is considered statistically significant.

## 3. Results

### 3.1. Identification and Enrichment Analysis of DEGs

A total of 14,689 DEGs in CD8+ T cells between vitiligo patients and normal controls were found among 33,002 background genes using the DESeq2 package in the R program with the following criteria: log FC (normalized fold change) > 1 or <-1 and adjusted *p* value < 0.001. A total of 1147 DEGs were further selected using more stringent criteria: currently annotated with a gene name, FPKM > 1, log FC > 2 or <-2, and adjusted *p* value < 0.001, including 373 upregulated genes and 774 downregulated genes in the vitiligo group compared to normal controls.

**Figure 1:**
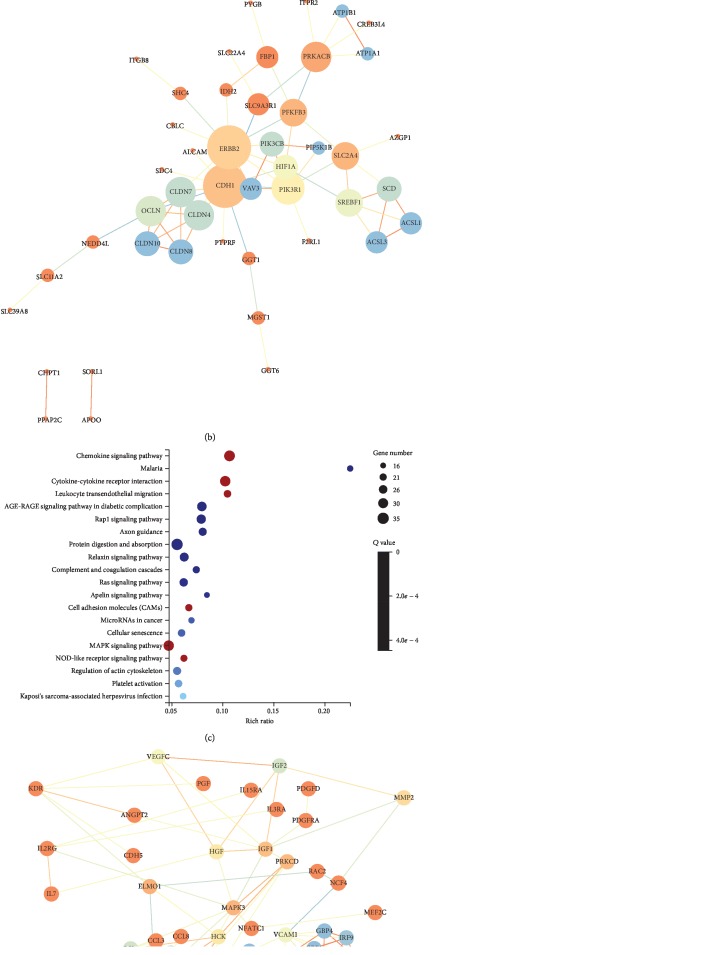
KEGG pathway enrichment analysis and PPI network. (a) The top 20 of all significant KEGG pathways for upregulated DEGs were selected (*p* < 0.05). Potentially related 12 pathways for upregulated DEGs in vitiligo were picked out and marked by red bubble. (b) PPI network for upregulated DEGs. (c) The top 20 of all significant KEGG pathways for downregulated DEGs were identified (*p* < 0.05). The most related 6 pathways for downregulated DEGs in vitiligo were figured out and marked by red bubble. (d) PPI network for downregulated DEGs.

To determine the relevant pathways, we performed KEGG pathway enrichment analysis for the DEGs. The top 20 of all significant KEGG pathways for upregulated and downregulated DEGs were selected (*p* < 0.05) (Figures 1(a) and 1(c)). Among these pathways, we further picked out 12 potential vitiligo-related pathways for upregulated DEGs and 6 for downregulated DEGs, respectively. Based on these specific pathways, 61 upregulated and 115 downregulated DEGs were finally screened out. Then, to investigate the interactions of the identified DEGs, we performed protein-protein interaction (PPI) analysis (STRING, http://www.string-db.org) (Figures 1(b) and 1(d)). Based on KEGG pathway enrichment analysis and PPI, we finally identified 16 upregulated and 23 downregulated genes (Table 1).

**Figure 2:**
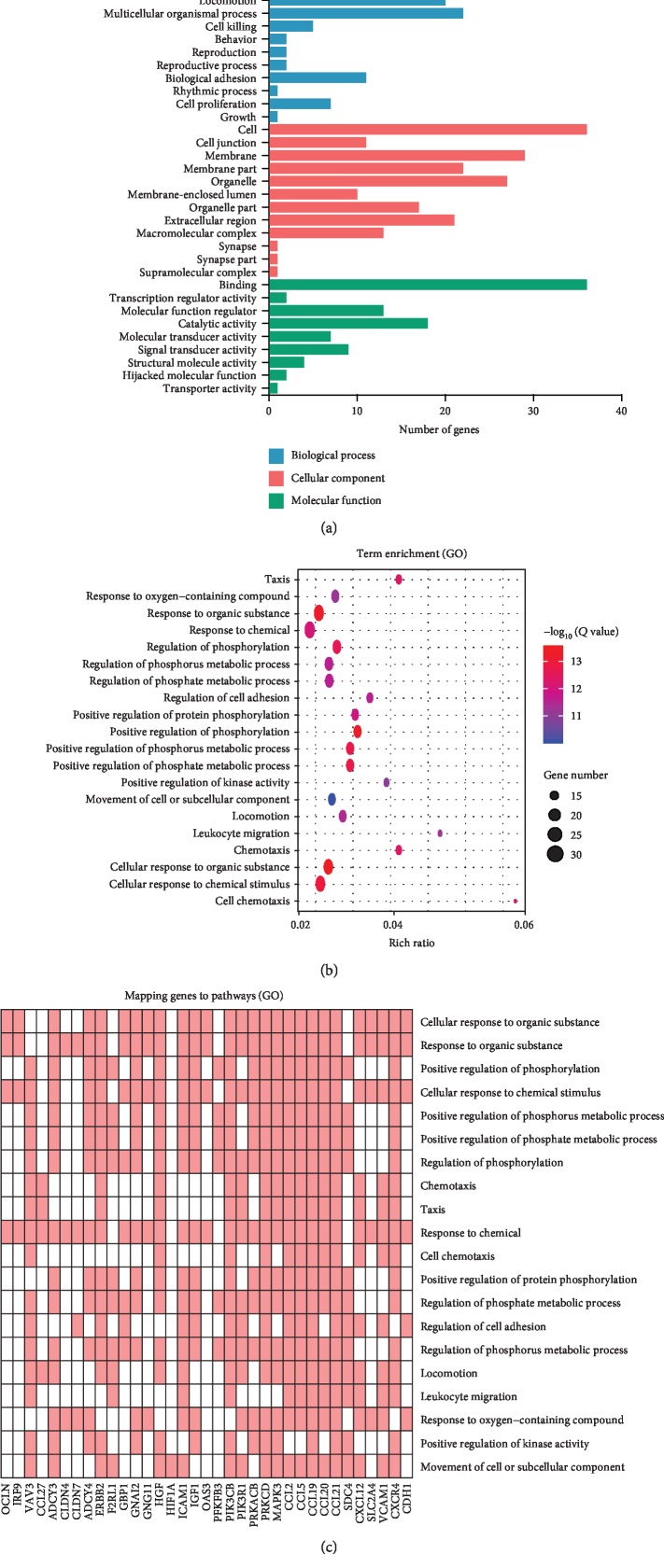
GO classification of the identified genes. (a) Biological process, cellular component, and molecular function of GO classification in the 39 DEGs. (b, c) GO biological process analysis of the 39 DEGs. Advanced bubble chart shows enrichment of DEGs in biological processes.

Then, the 39 DEGs were annotated by GO classification, and GO functional enrichment analysis was conducted to investigate their functions. The top 20 significantly enriched biology processes are shown in the GO term results for the 39 DEGs (adjusted values of *p* < 0.01) in Figures 2(a)–2(c). Some of these GO terms, such as response chemical, cell chemotaxis, regulation of cell adhesion, response to oxygen-containing compound, and movement of cell or subcellular component, are potentially related to the pathogenesis of vitiligo.

### 3.2. Validation of DEGs in CD8+ T Cells of Patients with Vitiligo

**Figure 3:**
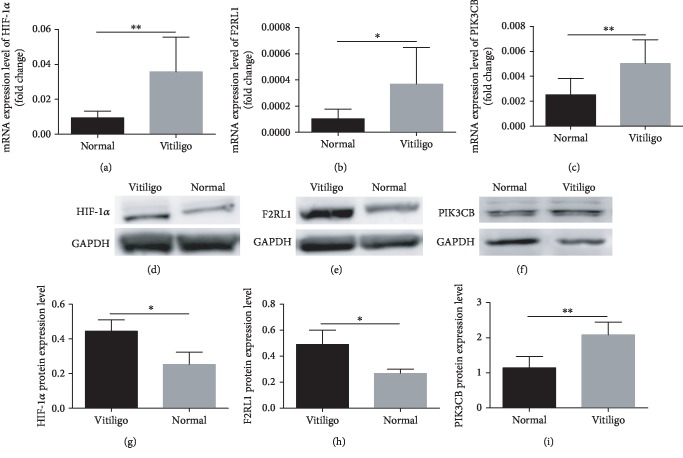
Validation of DEGs by RT-qPCR and western blotting. Among 3 identified genes, the mRNA expression levels of HIF-1*α* (*p* < 0.01) (a), F2RL1 (*p* < 0.05) (b), and PIK3CB (*p* < 0.01) (c) were elevated in CD8+ T cells from PBMCs of vitiligo patients (*n* = 8) compared to normal control (*n* = 8). (d–f) HIF-1*α* (*p* < 0.05) (*n* = 3), F2RL1 (*p* < 0.05) (*n* = 3), and PIK3CB (*p* < 0.01) (*n* = 4) were increased in CD8+ T cells from PBMCs of vitiligo patients compared to normal control. (g–i) Statistical analysis of HIF-1*α*, F2RL1, and PIK3CB was performed between vitiligo patients and normal control. Data are shown as mean ± SEM. ^∗^*p* < 0.05; ^∗∗^*p* < 0.01.

Among the 39 selected DEGs, mRNA expression levels were validated in CD8+ T cells separated from PBMCs by RT-qPCR. The results of the 3 genes were consistent with the RNA-sequencing (RNA-Seq) data. Among the 3 identified genes, the mRNA expression levels of PIK3CB (*p* < 0.01), F2RL1 (*p* < 0.05), and HIF-1*α* (*p* < 0.01) were increased in vitiligo patients (Figures 3(a)–3(c)).

To further confirm our findings, we validated the protein expression levels of these 3 DEGs in CD8+ T cells from PBMCs by western blotting. We found that F2RL1 (*p* < 0.05), PIK3CB (*p* < 0.01), and HIF-1*α* (*p* < 0.05) were significantly increased in vitiligo patients compared to normal controls, which was consistent with the results of qRT-PCR (Figures 3(d)–3(i)).

**Figure 4:**
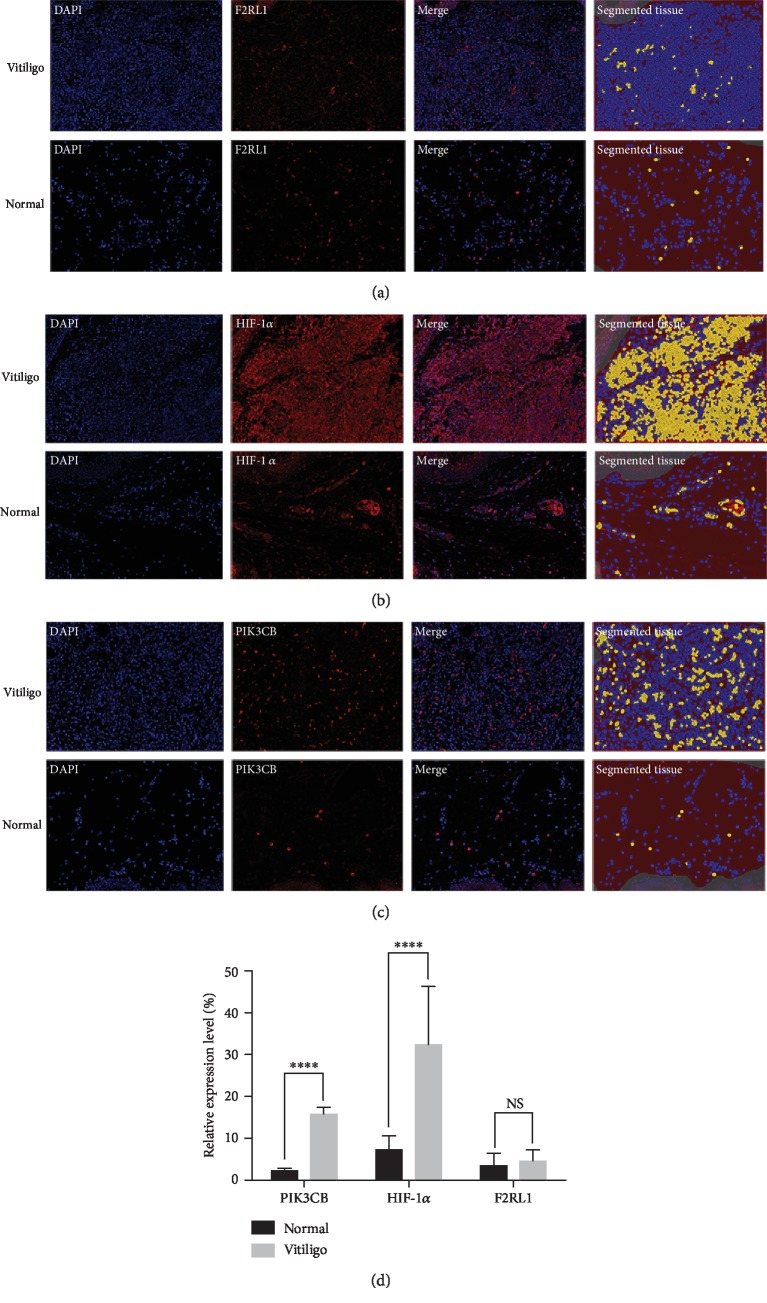
Opal IHC validation of DEGs. F2RL1 (a) were nonsignificant in lesional skin of patients with vitiligo compared with normal control (*n* = 10). HIF-1*α* (b) and PIK3CB (c) were significantly elevated in vitiligo (*n* = 10). Three or more areas were randomly selected. Pictures were taken at ×200 and analyzed using inFORM image analysis software, which quantifies the segmented tissue based on respective positive expression (yellow). (d) Data are shown as mean ± SEM. ^∗∗∗∗^*p* < 0.0001. NS = nonsignificant.

We also investigated the protein expression levels of 3 DEGs in lesional skin of patients with vitiligo. Notably, we found that HIF-1*α* (*p* < 0.0001) and PIK3CB (*p* < 0.0001) were significantly increased in vitiligo. Nevertheless, F2RL1 presented a nonsignificant elevated expression level (*p* = 0.45) in vitiligo compared with normal controls (Figures 4(a)–4(d)).

### 3.3. Specific CG Sites of HIF-1*α* Were Hypomethylated in CD8+ T Cells from PBMCs of Patients with Vitiligo

**Figure 5:**
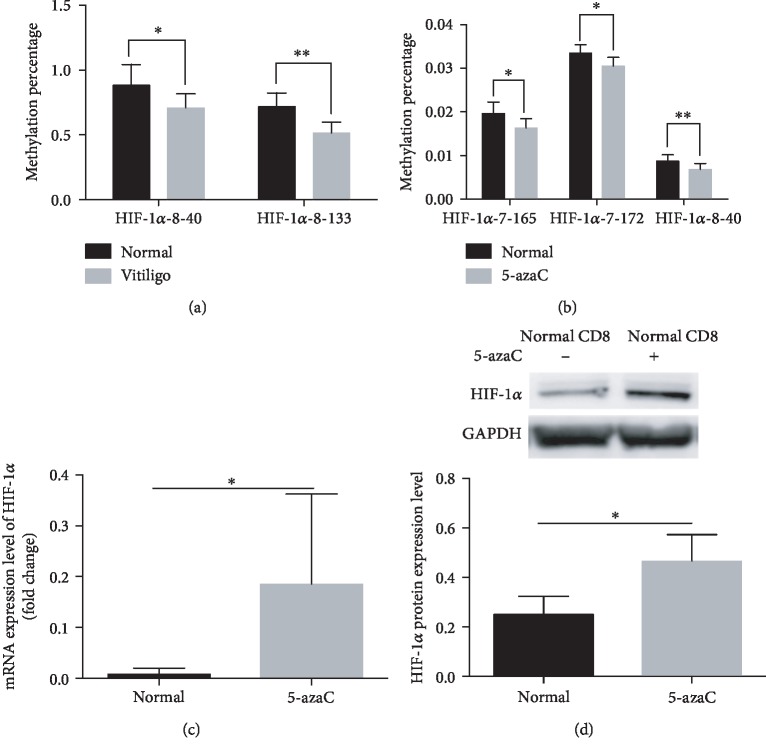
HIF-1*α* promoter methylation patterns in CD8+ T cells from vitiligo patients, 5-azaC-treated CD8+ T cells, and normal controls. (a) Methylation status of 2 CG pairs (positions HIF-1*α*-8-40 and HIF-1*α*-8-133) was decreased in vitiligo patients compared to these positions of normal control (*p* < 0.05 and *p* < 0.01, respectively) (*n* = 6). (b) Methylation status of 3 CG pairs (positions HIF-1*α*-7-165, HIF-1*α*-7-172, and HIF-1*α*-8-40) was reduced in 5-azaC-treated CD8+ T cells compared to normal CD8+ T cells (*p* < 0.05, *p* < 0.05, and *p* < 0.01, respectively) (*n* = 6). (c, d) The mRNA and protein expression level of HIF-1*α* (*p* < 0.05 and *p* < 0.05, respectively) was increased in 5-azaC-treated CD8+ T cells compared to normal CD8+ T cells (*n* = 8 and *n* = 3, respectively). Data are shown as mean ± SEM. ^∗^*p* < 0.05; ^∗∗^*p* < 0.01.

To investigate the mechanisms of the overexpression of HIF-1*α* and F2RL1 in CD8+ T cells in vitiligo, the methylation status of 47 CG pairs in the HIF-1*α* gene promoter (from +737 to -175) and 30 CG pairs in the F2RL1 gene promoter (from +711 to -238) with respect to the transcriptional start site (TSS) was analyzed in vitiligo- and normal-CD8+ T cells from PBMCs by using MethylTarget (Supplemental [Supplementary-material supplementary-material-1]). Ultimately, we found that two CpG sites of the HIF-1*α* promoter were hypomethylated (vitiligo vs. normal control: HIF-1*α*-8-40 0.67% (0.48%-0.88%) vs. 0.87% (0.75%-1.08%), *p* < 0.05, and HIF-1*α*-8-133 0.54% (0.48%-0.88%) vs. 0.75% (0.61%-0.89%), *p* < 0.01, respectively) in CD8+ T cells of vitiligo compared to normal controls (Figure 5(a)). However, the DNA methylation levels of the F2RL1 promoter showed non significance in vitiligo patients compared to controls.

### 3.4. Treatment of Normal CD8+ T Cells with 5-azaC Enhances mRNA and Protein Expression of HIF-1*α*

Previous studies have suggested that 5-azaC-treated CD4+ T cells decrease the DNA methylation level of some DNA methylation-sensitive genes, such as CD11a [36], CD70 [24], perforin [37], IL10, and IL13 [38]. To further test whether the overexpressed HIF-1*α* is caused by the DNA hypomethylation, normal CD8+ T cells were treated with 1 *μ*M 5-azaC for 72 h. Intriguingly, we found that the CpG sites of HIF-1*α*-8-40 in 5-azaC-treated-normal CD8+ T cells were hypomethylated compared to untreated-normal CD8+ T cells (0.68% (0.51%-0.83%) vs. 0.87% (0.75%-1.08%), *p* < 0.01), which is consistent with the results of vitiligo CD8+ T cells. In addition, the DNA methylation levels of HIF-1*α*-7-165 and HIF-1*α*-7-172 were also decreased in 5-azaC-treated CD8+ T cells than normal CD8+ T cells (1.6% (1.5%-2.0%) vs. 2.0% (1.7%-2.2%), *p* < 0.05, and 3.0% (2.9%-3.4%) vs. 3.4% (3.2%-3.5%), *p* < 0.05, respectively). Then, we investigated the mRNA and protein expression levels of HIF-1*α*, and we found that the mRNA and protein levels were both increased in the 5-azaC-treated-normal CD8+ T cells (*p* < 0.05 and *p* < 0.05, respectively) compared to untreated-normal CD8+ T cells (Figures 5(b)–5(d)).

## 4. Discussion

Autoimmunity is an important pathogenic factor in vitiligo. Autoimmune cells migrating from peripheral blood to the skin can directly or indirectly mediate the destruction of melanocytes [39, 40]. Elevated CD8+ T cells have been found in both the lesional skin and peripheral blood of vitiligo patients [4–6]. A cytotoxic role for activated CD8+ T cells mediating the destruction of melanocytes has been well established *in vivo* and *in vitro* [7, 10, 41, 42]. In order to decipher the intricate interplay of cellular processes and molecular mechanisms in the pathogenesis of vitiligo, previous studies using transcriptome sequencing have focused on the whole lesional skin and peripheral blood [43–45]. Nevertheless, despite the critical pathogenic role of CD8+ T cells in vitiligo, it is difficult to clarify their precise molecular mechanisms via transcriptome sequencing of whole lesional skin and peripheral blood due to the existence of other cells, such as keratinocytes, fibroblasts, CD4+ T cells, B cells, and macrophages. To date, the factors that drive activation, proliferation, and the cytotoxic functions of melanocyte-specific cytotoxic CD8+ T cells remain obscure. To our knowledge, the present study is the first to provide transcriptomic analysis of CD8+ T cells from lesional skin in vitiligo. Three DEGs (HIF-1*α*, F2RL1, and PIK3CB) were identified and further investigated by RT-qPCR.

The HIF-1*α* gene was identified as being upregulated in vitiligo for the first time in this study. HIF-1*α* is an oxygen-sensitive transcription factor that functions as a master regulator of the cellular and systemic homeostatic response to hypoxia [46, 47]. Importantly, HIF-1*α* acts as a critical regulator in both innate and adaptive immunities [48, 49]. For innate immunity, HIF-1*α* can increase cell motility and the expression levels of proinflammatory cytokines in macrophages [50, 51]. HIF-1*α* also functions as an important regulator for myeloid cells against bacterial infection by producing antimicrobial peptides, TNF-*α*, and nitric oxide [50, 51]. For adaptive immunity, HIF-1*α* has been found to play a crucial role in the development and effector functions of immune cells, such as Treg cells, Th17 cells, B cells, and dendritic cells via regulating the production of Foxp3, IL-17, IL-10, TNF-*α*, and IL-6, respectively [47, 52]. Accumulating studies have demonstrated that HIF-1*α* is significantly elevated in a lupus murine model, rheumatoid arthritis patient synovial fluids, and psoriatic lesions and peripheral blood via the secretion of proinflammatory cytokines (IL-17, IL-33, and IL-6, respectively) to mediate the inflammation and immune response [52–56]. Importantly, Doedens et al. provided evidence that the increased HIF-1*α* promotes glycolytic metabolism, enhances cytotoxicity via the secretion of perforin and granzyme B, and activates more costimulatory and inhibitory receptors in CD8+ T cells during chronic infection and melanoma [57]. In the present study, HIF-1*α* was increased in CD8+ T cells of vitiligo lesions and PBMCs, which is consistent with the results of typical autoimmune diseases. Based on the above evidence, elevated HIF-1*α* may mediate immune responses by inducing the generation of proinflammatory cytokines in CD8+ T cells and reinforce their cytotoxicity by secreting cytotoxic granules to destruct melanocytes in vitiligo. Nevertheless, it is necessary to elucidate this in further functional studies.

Environmental factors (ultraviolet irradiation, chemical exposure (monobenzone), and trauma) act as crucial exogenous factors to produce oxidative byproducts, such as reactive oxygen species (ROS) in vitiligo [5, 6, 58]. ROS-induced oxidative stress is believed to contribute to the generation, activation, and recruitment of autoreactive CD8+ T cells in vitiligo [16, 59]. Moreover, it is clear that DNA methylation is the most important epigenetic process, which refers to environment-induced changes in gene expression [3, 60]. It is generally accepted that DNA demethylation promotes gene transcription and activation [61, 62]. In this study, we have demonstrated that the mRNA and protein expression levels of HIF-1*α* are elevated in vitiligo. To further investigate the mechanisms of the overexpression of HIF-1*α* in vitiligo CD8+ T cells, we confirmed the DNA methylation status of the HIF-1*α* promoter in vitiligo patients. The results showed that two specific CpG sites of the HIF-1*α* promoter were hypomethylated in patients with vitiligo. Consistently, DNA methylation levels of specific CpG sites in the HIF-1*α* promoter were decreased in DNA methyltransferase inhibitor 5-azaC-treated CD8+ T cells. Furthermore, accompanied by the DNA hypomethylation, we found that mRNA and protein expression levels of HIF-1*α* were both significantly increased in 5-azaC-treated CD8+ T cells. Thus, we suggest that the higher levels of HIF-1*α* are related to its DNA hypomethylation in vitiligo. Intriguingly, previous studies have shown that ROS can mediate the transcriptional and translational regulation of HIF-1*α* [63, 64]. A plethora of studies indicate that ROS is involved in the aberrant DNA methylation levels of numerous genes [65–67]. Based on this, we speculated that elevated HIF-1*α* may be caused by environmental factor-induced ROS via DNA hypomethylation mechanism, which may further promote the activation of CD8+ T cells in vitiligo. Nonetheless, how ROS influences HIF-1*α* through DNA hypomethylation and how the overexpressed HIF-1*α* acts on the functions of CD8+ T cells remain to be confirmed.

F2R-Like Trypsin Receptor 1 (F2RL1), also called protease-activated receptor 2 (PAR2), is a G-protein-coupled receptor that mediates cellular responses via the activation of heteromeric G-proteins [68]. F2RL1 is expressed in most immune cells, such as neutrophils [69], eosinophils [70], monocytes [71], macrophages [72], DCs [72], mast cells [73], and T cells [74, 75]. In the skin, F2RL1 is mainly expressed in keratinocytes [76]. Oxidative stress can induce the dysfunction of keratinocytes, and then, stressed keratinocytes secrete CXCL16 to recruit CD8+ T cells to lesional sites to promote vitiligo [77]. In addition, Hou et al. have provided evidence that in keratinocytes, the activation of F2RL1 results in the secretion of IL-8/CXCL8, which can promote the recruitment of T cells to aggravate inflammation [76]. In the current study, the mRNA and protein expression levels of F2RL1 were increased in CD8+ T cells from PBMCs in vitiligo. Given that F2RL1 is also a DNA methylation-sensitive gene, we also validated the DNA methylation status of the F2RL1 promoter to investigate the mechanism of the overexpression of F2RL1 in CD8+ T cells from PBMCs in vitiligo patients. However, the DNA methylation level of the F2RL1 promoter showed no variation in vitiligo patients. Based on the above, we speculated that aberrantly elevated F2RL1 may be caused by oxidative stress-related cells that secrete chemokines to attract circulating CD8+ T cell migrating to the vitiligo lesional regions. However, this awaits further investigations.

Phosphoinositide 3-kinases (PI3Ks) are important intracellular signaling molecules that regulate multiple signaling pathways associated with cellular growth, survival, proliferation, and metabolism [78, 79]. PIK3CB/p100*β* is a catalytic subunit of the PI3K family [79]. Evidence has demonstrated that PIK3CB is expressed in immune cells, but its roles are not well understood [80]. PIK3CB plays a crucial role in ROS production through neutrophils by recognizing immobilized immune complexes. PIK3CB is implicated in cellular responses to promote inflammation [81], and the alleviation of inflammation mediated by immune complexes is found in p110*β*-deficient mice [80]. In this study, we found that the mRNA and protein expression level of PIK3CB was significantly increased in vitiligo. Due to the unambiguous role of PIK3CB in the production of ROS, we hypothesized that oxidative stress may contribute to the cytotoxic functions of CD8+ T cells to initiate vitiligo via PIK3CB. However, this molecular mechanism in vitiligo warrants further support. Interestingly, studies have revealed an interaction between PI3K and HIF-1*α*, and the activation of the PI3K/AKT pathway correlates with ROS-mediated transcriptional and translational regulation of HIF-1*α* [64, 82]. In addition, Huang et al. have reported that KC7F2 (a HIF-1*α* inhibitor) downregulates the levels of hypoxia-induced inflammatory cytokines in human monocytic cells (THP-1 cells) through the inhibition of the PI3K/Akt pathway [83]. In our study, the expression levels of PIK3CB and HIF-1*α* were both increased in the patients with vitiligo. Therefore, we hypothesize that HIF-1*α* may be involved in the cytotoxic functions of CD8+ T cells via the PI3K/Akt pathway. However, studies regarding the exact mechanisms about the interplay between PIK3CB and HIF-1*α* in vitiligo still need to be extended.

In conclusion, this study is the first study to use the transcriptome sequencing of CD8+ T cells from the lesional skin of vitiligo. We identified the significant DEGs from the transcriptome analysis results and further verified their mRNA and protein expression levels. Ultimately, we found that the mRNA and protein levels of HIF-1*α*, F2RL1, and PIK3CB genes were all elevated in the CD8+ T cells in vitiligo. Importantly, CD8+ T cells that migrate to inflammation sites to successfully kill melanocytes need two major steps: activation and chemotaxis. In the present study, we identified three DEGs (HIF-1*α*, F2RL1, and PIK3CB) that are all closely related to ROS. Intriguingly, these three DEGs not only were correlated with the activation of CD8+ T cells but also played an important role in CD8+ T cell migration in vitiligo. However, additional studies are needed to clarify the exact roles of these three DEGs and their mutual effects in CD8+ T cell activation and migration in vitiligo.

## Figures and Tables

**Table 1:** Top 39 DEGs with most significant *p* value and fold change.

Gene	log2(HN/FK)	*Q* value (FK-vs-HN)	Trend
OCLN	3.132628422	<0.001	Up
CLDN8	4.606907309	<0.001	Up
CLDN10	6.188293528	<0.001	Up
CLDN4	5.014616872	<0.001	Up
CLDN7	6.115564414	<0.001	Up
CDH1	2.803771269	<0.001	Up
PIK3R1	2.265781754	<0.001	Up
ERBB2	2.928337457	<0.001	Up
PIK3CB	2.083538414	<0.001	Up
SLC2A4	2.060961012	<0.001	Up
PRKACB	2.768390347	<0.001	Up
HIF1A	2.049673199	<0.001	Up
PFKFB3	2.355903765	<0.001	Up
SDC4	2.331146517	<0.001	Up
F2RL1	2.039230759	<0.001	Up
VAV3	3.088326822	<0.001	Up
CXCR4	-2.049867246	<0.001	Down
GNAI2	-2.666675949	<0.001	Down
CCL20	-2.892742759	<0.001	Down
GNG11	-2.057636004	<0.001	Down
GNG2	-2.792026453	<0.001	Down
CCL27	-2.530145696	<0.001	Down
CCL21	-4.852282333	<0.001	Down
CCL19	-6.40245659	<0.001	Down
ADCY3	-3.530651817	<0.001	Down
ADCY4	-2.038698263	<0.001	Down
CXCL12	-3.37944294	<0.001	Down
CCL5	-4.649859535	<0.001	Down
CCL2	-3.763197581	<0.001	Down
ICAM1	-2.820107258	<0.001	Down
VCAM1	-2.396227235	<0.001	Down
GBP1	-2.459306784	<0.001	Down
GBP4	-2.340546416	<0.001	Down
IRF9	-2.008373387	<0.001	Down
OAS3	-2.747670137	<0.001	Down
PRKCD	-2.324853772	<0.001	Down
MAPK3	-3.132412248	<0.001	Down
IGF1	-3.869011486	<0.001	Down
HGF	-2.546020974	<0.001	Down

## Data Availability

The data generated and analyzed during this study are available upon reasonable request from the corresponding author.

## Supplementary Materials

Supplementary Materials Figure S1: details of the transcriptome sequencing of CD8+ T cells from the vitiligo lesional skin and normal controls. (a, b) Correlation heat map and principal components for CD8+ T cells between vitiligo lesional skin and normal controls. (c) Clustering heat map of DEGs in CD8+ T cells between vitiligo lesional skin and normal controls. The *x*-axis represents the sample. The *y*-axis represents the DEGs. ^∗^V = vitiligo; NC = normal controls. Figure S2: validation of 36 DEGs by RT-qPCR. NS = nonsignificance. Table S1: the list of primers and reference sequences in RT-qPCR. Table S2: the list of primers and reference sequences in MethylTarget. Table S3: details of CpG regions in the HIF-1*α* and F2RL1 promoter.
