# Supplementary material for: Transcriptome Analysis and Emerging Driver Identification of CD8+ T Cells in Patients with Vitiligo
Source: Oxid Med Cell Longev. 2019 Nov 26;2019:2503924. doi: 10.1155/2019/2503924 (PMC6899274; doi:10.1155/2019/2503924)
Supplement: Supplementary Materials — Figure S1: details of the transcriptome sequencing of CD8+ T cells from the vitiligo lesional skin and normal controls. (a, b) Correlation heat map and principal components for CD8+ T cells between vitiligo lesional skin and normal controls. (c) Clustering heat map of DEGs in CD8+ T cells between vitiligo lesional skin and normal controls. The x-axis represents the sample. The y-axis represents the DEGs. ∗V = vitiligo; NC = normal controls. Figure S2: validation of 36 DEGs by RT-qPCR. NS = nonsignificance. Table S1: the list of primers and reference sequences in RT-qPCR. Table S2: the list of primers and reference sequences in MethylTarget. Table S3: details of CpG regions in the HIF-1α and F2RL1 promoter. [file 2503924.f1.pdf]

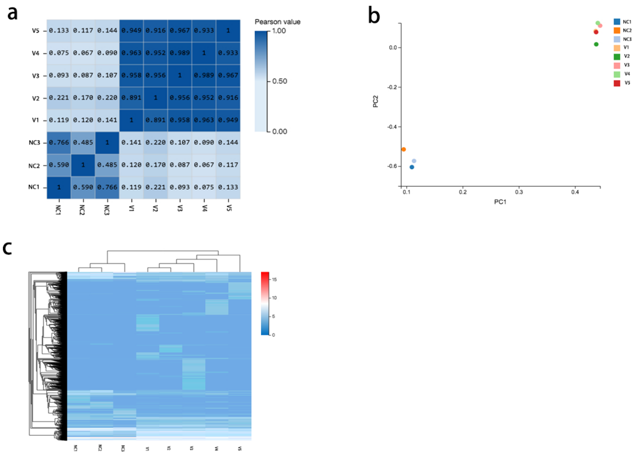


Figure S1: Details of the transcriptome sequencing of CD8+ T cells from the vitiligo lesional skin and normal controls. a-b. Correlation heatmap and principal components for CD8+ T cells between vitiligo lesional skin and normal controls. c. Clustering heatmap of DEGs in CD8+ T cells between vitiligo lesional skin and normal controls. The X-axis represents the sample. The Y-axis represents the DEGs. *V=Vitiligo, NC=Normal controls.

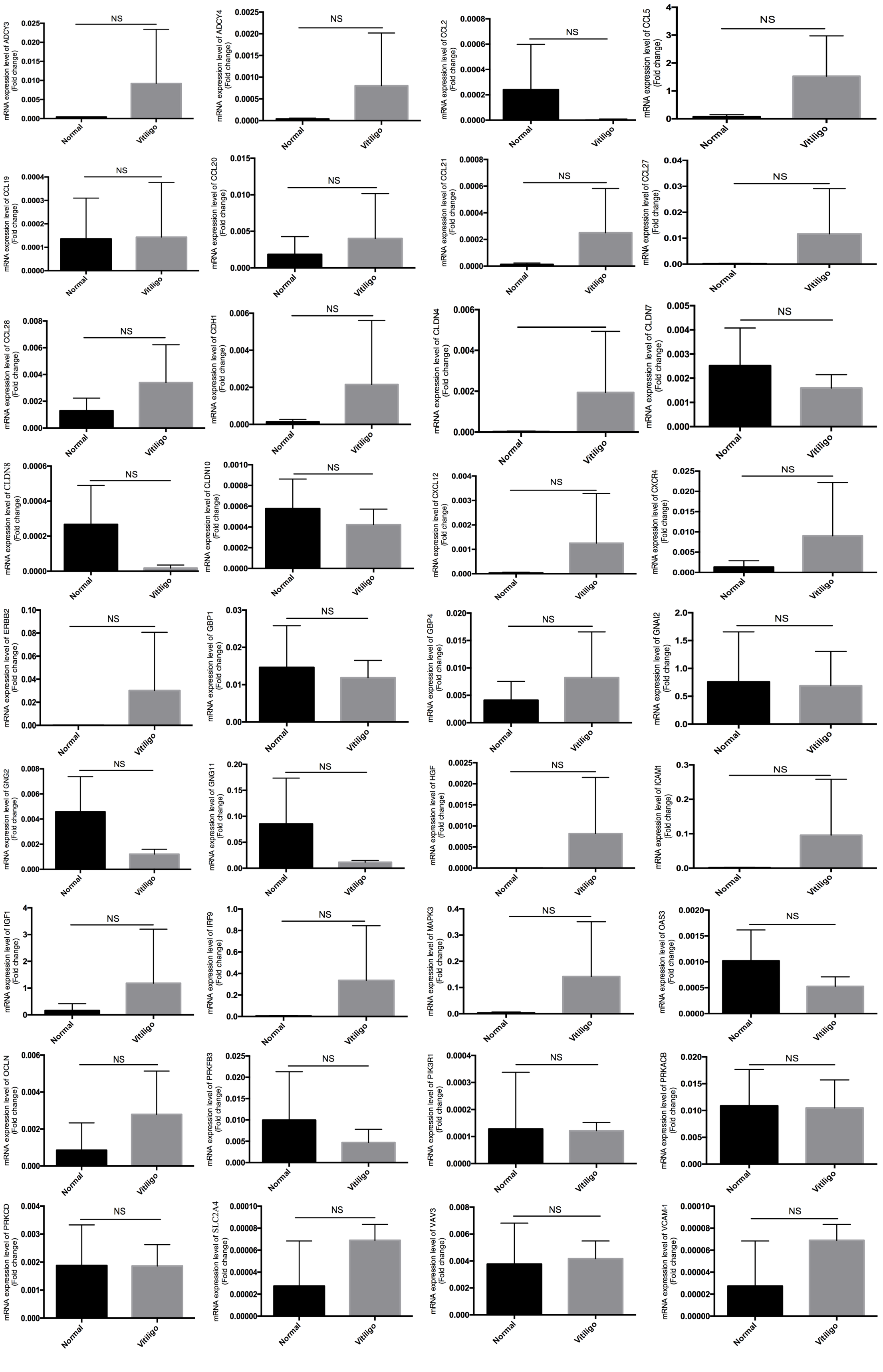


Figure S2: Validation of 36 DEGs by RT-qPCR. NS=non-significance.

Table S1. The list of primers and reference sequences in RT-qPCR

| Primer name | Primer sequence (5’ to 3’) |
|---|---|
| ADCY3-F | GGACCAGCAGCAGTTCAA |
| ADCY3-R | AAGCCGCAGATGCAGTAG |
| CCL2-F | TAGCAGCCACCTTCATTC |
| CCL2-R | CTTCTTTGGGACACTTGC |
| CCL5-F | AGCCCTCGCTGTCATCCT |
| CCL5-R | GGCAATGTAGGCAAAGCAG |
| CCL19-F | TTCTCATCAAGGATGGCT |
| CCL19-R | CTCTGGATGATGCGTTCTA |
| CCL20-F | ACCGTATTCTTCATCCTAA |
| CCL20-R | TTCACCCAAGTCTGTTTT |
| CCL21-F | GGAAGATTCCCGCCAAGG |
| CCL21-R | AGCTGCTGCACCCAGAGC |
| CCL27-F | TCCTGAGCCCAGACCCTA |
| CCL27-R | GCAGTTCCACCTGGATGAC |
| CCL28-F | GCTCCTGGAAAGAGTGAATATGTG |
| CCL28-R | GTATGGTTGTGCGGGCTGAC |
| CDH1-F | CTTGGTCTACGCCTGGGACT |
| CDH1-R | TTGGGAAATGTGAGCAATTCTG |
| CLDN4-F | CCTTCATCGGCAGCAACA |
| CLDN4-R | CAGCAGCGAGTCGTACACCTT |
| CXCL12-F | AAGGTCGTGGTCGTGCTG |
| CXCL12-R | CCACTTTAGCTTCGGGTCA |
| CXCR4-F | TGACACTCCCGCCCAATA |
| CXCR4-R | CCCTGAGCCCATTTCCTC |
| ADCY4-F | TGCTAGTGACAAACAGGCCC |
| ADCY4-R | GGACACATTGGGAGCTTGGA |
| ERBB2-F | CAGAGGCTGCGGATTGTG |
| ERBB2-R | TGTTCAGCGGGTCTCCAT |
| F2RL1-F | GAAACTTAAAATTCAGGAGCAA |
| F2RL1-R | GAAAACTCATCCACAGAAAAGA |

| | |
|---|---|
| GBP1-F | TGACATACTCCCTGAAGCTG |
| GBP1-R | GGCCGATCAAAGACAAAG |
| GBP4-F | TTCTCAGCCCGTGGTGGT |
| GBP4-R | TGGAGCCCAGAGGGAAGC |
| HGF-F | GCCATGAATTTGACCTCTA |
| HGF-R | GCTGACATTTGATGCCACT |
| HIF1A-F | TACCATCAGCTATTTGCGTG |
| HIF1A-R | GAGAACCATAACAAAACCATCC |
| ICAM1-F | CTGCTCGGGGCTCTGTTC |
| ICAM1-R | AGGAGGTGCTGCATGTCA |
| IGF1-F | TGCCTGCTCACCTTCACC |
| IGF1-R | CCATACCCTGTGGGCTTGT |
| IRF9-F | CAGGCAAGCAGGACTTCC |
| IRF9-R | GGCGAGTCTTCCAGACAG |
| MAPK3-F | GGAGGTGGAGATGGTGAAGG |
| MAPK3-R | GGGCTGATCTTCTTGATGGC |
| OAS3-F | GCGGGTGCTGAAAACTGT |
| OAS3-R | GCAGTCGAGGAAGATGACAA |
| OCLN-F | CAATGGCAAAGTGAATGAC |
| OCLN-R | CAGGCGAAGTTAATGGAA |
| PFKFB3-F | CTGACTCGCTACCTCAACTGG |
| PFKFB3-R | CGGACTTTCATGGCTTCCT |
| PIK3CB-F | ACCCAGGGGAAAAATTAGAC |
| PIK3CB-R | ACAATCCCACAAGTGACAGG |
| PIK3R1-F | ACATTGCCCCGCCTCTTCTTAT |
| PIK3R1-R | TGTCGTAATTCTGCCAGGTTGC |
| PRKACB-F | TAGCCAAAGCCAAAGAAGACTT |
| PRKACB-R | TGACTCTTCCAAATGAACCTGT |
| PRKCD-F | AGAAGAACAATGGCAAGGC |
| PRKCD-R | CTCCAGGAAATACTGAACAGAC |
| SLC2A4-F | ACTGGCCATTGTTATCGGCA |
| SLC2A4-R | GTCAGGCGCTTCAGACTCTT |
| CLDN8-F | GTGGATGAATTGCGTGAGGC |

| | |
|---|---|
| CLDN8-R | TAGGTCCGGAGAAAGAGCCA |
| CLDN10-F | GGAGGCTCCGATAAAGCCAA |
| CLDN10-R | GCACAGCCCTGACAGTATGA |
| CLDN7-F | TTTCATCGTGGCAGGTCTTG |
| CLDN7-R | TGTTGGTAGGGATCAAAGGGT |
| GNG11-F | CGAAAATGCCTGCCCTTCAC |
| GNG11-R | TAGACACTTGTTGTCTCTGCAAC |
| GNAI2-F | TCAACGACTCAGCTGCCTAC |
| GNAI2-R | CATGCATGCGGTTCATCTCC |
| GNG2-F | GGAGCTAAGATCTGCCAGTGA |
| GNG2-R | TCAGGCCCCTCTCAAAGACT |
| VAV3-F | CACGACTTTCTCGAACACCTAT |
| VAV3-R | GGCCTTTGTAGATGTCTTCATC |
| VCAM-1-F | TCCGTCTCATTGACTTGC |
| VCAM-1-R | TGACTGTGATCGGCTTCC |

Table S2. The list of primers and reference sequences in MethylTarget

| Primer Name | Primer Sequence |
|---|---|
| HIF-1α_6F | GGTTGGGTTTTGATAAGTTATTTGAG |
| HIF-1α_6R | ATATCTTCACRACRAACRACCCCCAAACTC |
| HIF-1α_7F | GGGTTATAGAGTAGGTTGGGTTTT |
| HIF-1α_7R | CTCCCCTCCCCTCTCTTC |
| HIF-1α_8F | GAGGTGGAGGYGGGTTTTT |
| HIF-1α_8R | CACCCCCATCTCCTTTCTCTT |
| F2RL1_4F | TTTTTATAGTTAGGAAAGGTAAGGGAGA |
| F2RL1_4R | CCCCACCCCCTTCCCTAC |
| F2RL1_5F | AAGAAAGGTTTTTAGTAGTTGGAGAGG |
| F2RL1_5R | CCTAATCCCCTCCCCCAACT |

Table S3. Details of CpG regions in HIF-1α and F2RL1 promoter

| Target | Position | Chr* | Genome Position | Distance 2TSS* | Type |
|---|---|---|---|---|---|
| HIF1A_6 | 35 | 14 | 62162334 | 216 | CG |
| HIF1A_6 | 41 | 14 | 62162340 | 222 | CG |
| HIF1A_6 | 47 | 14 | 62162346 | 228 | CG |
| HIF1A_6 | 54 | 14 | 62162353 | 235 | CG |
| HIF1A_6 | 57 | 14 | 62162356 | 238 | CG |
| HIF1A_6 | 64 | 14 | 62162363 | 245 | CG |
| HIF1A_6 | 68 | 14 | 62162367 | 249 | CG |
| HIF1A_6 | 86 | 14 | 62162385 | 267 | CG |
| HIF1A_6 | 98 | 14 | 62162397 | 279 | CG |
| HIF1A_6 | 103 | 14 | 62162402 | 284 | CG |
| HIF1A_6 | 142 | 14 | 62162441 | 323 | CG |
| HIF1A_6 | 144 | 14 | 62162443 | 325 | CG |
| HIF1A_6 | 161 | 14 | 62162460 | 342 | CG |
| HIF1A_6 | 170 | 14 | 62162469 | 351 | CG |
| HIF1A_6 | 175 | 14 | 62162474 | 356 | CG |
| HIF1A_7 | 30 | 14 | 62162855 | 737 | CG |
| HIF1A_7 | 42 | 14 | 62162843 | 725 | CG |
| HIF1A_7 | 45 | 14 | 62162840 | 722 | CG |
| HIF1A_7 | 47 | 14 | 62162838 | 720 | CG |
| HIF1A_7 | 58 | 14 | 62162827 | 709 | CG |
| HIF1A_7 | 62 | 14 | 62162823 | 705 | CG |
| HIF1A_7 | 65 | 14 | 62162820 | 702 | CG |
| HIF1A_7 | 92 | 14 | 62162793 | 675 | CG |
| HIF1A_7 | 111 | 14 | 62162774 | 656 | CG |
| HIF1A_7 | 116 | 14 | 62162769 | 651 | CG |
| HIF1A_7 | 132 | 14 | 62162753 | 635 | CG |
| HIF1A_7 | 143 | 14 | 62162742 | 624 | CG |
| HIF1A_7 | 146 | 14 | 62162739 | 621 | CG |
| HIF1A_7 | 155 | 14 | 62162730 | 612 | CG |

| | | | | | |
|---|---|---|---|---|---|
| HIF1A_7 | 165 | 14 | 62162720 | 602 | CG |
| HIF1A_7 | 169 | 14 | 62162716 | 598 | CG |
| HIF1A_7 | 172 | 14 | 62162713 | 595 | CG |
| HIF1A_8 | 20 | 14 | 62162078 | -40 | CG |
| HIF1A_8 | 33 | 14 | 62162065 | -53 | CG |
| HIF1A_8 | 37 | 14 | 62162061 | -57 | CG |
| HIF1A_8 | 40 | 14 | 62162058 | -60 | CG |
| HIF1A_8 | 46 | 14 | 62162052 | -66 | CG |
| HIF1A_8 | 69 | 14 | 62162029 | -89 | CG |
| HIF1A_8 | 72 | 14 | 62162026 | -92 | CG |
| HIF1A_8 | 93 | 14 | 62162005 | -113 | CG |
| HIF1A_8 | 117 | 14 | 62161981 | -137 | CG |
| HIF1A_8 | 124 | 14 | 62161974 | -144 | CG |
| HIF1A_8 | 127 | 14 | 62161971 | -147 | CG |
| HIF1A_8 | 133 | 14 | 62161965 | -153 | CG |
| HIF1A_8 | 139 | 14 | 62161959 | -159 | CG |
| HIF1A_8 | 152 | 14 | 62161946 | -172 | CG |
| HIF1A_8 | 155 | 14 | 62161943 | -175 | CG |
| F2RL1_4 | 30 | 5 | 76114563 | -238 | CG |
| F2RL1_4 | 33 | 5 | 76114566 | -235 | CG |
| F2RL1_4 | 38 | 5 | 76114571 | -230 | CG |
| F2RL1_4 | 74 | 5 | 76114607 | -194 | CG |
| F2RL1_4 | 76 | 5 | 76114609 | -192 | CG |
| F2RL1_4 | 102 | 5 | 76114635 | -166 | CG |
| F2RL1_4 | 113 | 5 | 76114646 | -155 | CG |
| F2RL1_4 | 151 | 5 | 76114684 | -117 | CG |
| F2RL1_4 | 161 | 5 | 76114694 | -107 | CG |
| F2RL1_4 | 165 | 5 | 76114698 | -103 | CG |
| F2RL1_4 | 171 | 5 | 76114704 | -97 | CG |
| F2RL1_4 | 178 | 5 | 76114711 | -90 | CG |
| F2RL1_4 | 197 | 5 | 76114730 | -71 | CG |
| F2RL1_4 | 199 | 5 | 76114732 | -69 | CG |
| F2RL1_4 | 205 | 5 | 76114738 | -63 | CG |

| | | | | | |
|---|---|---|---|---|---|
| F2RL1_4 | 212 | 5 | 76114745 | -56 | CG |
| F2RL1_4 | 219 | 5 | 76114752 | -49 | CG |
| F2RL1_4 | 227 | 5 | 76114760 | -41 | CG |
| F2RL1_5 | 30 | 5 | 76115626 | 825 | CG |
| F2RL1_5 | 55 | 5 | 76115601 | 800 | CG |
| F2RL1_5 | 60 | 5 | 76115596 | 795 | CG |
| F2RL1_5 | 62 | 5 | 76115594 | 793 | CG |
| F2RL1_5 | 79 | 5 | 76115577 | 776 | CG |
| F2RL1_5 | 91 | 5 | 76115565 | 764 | CG |
| F2RL1_5 | 102 | 5 | 76115554 | 753 | CG |
| F2RL1_5 | 114 | 5 | 76115542 | 741 | CG |
| F2RL1_5 | 120 | 5 | 76115536 | 735 | CG |
| F2RL1_5 | 122 | 5 | 76115534 | 733 | CG |
| F2RL1_5 | 131 | 5 | 76115525 | 724 | CG |
| F2RL1_5 | 144 | 5 | 76115512 | 711 | CG |

*Chr=chromosome, TSS= transcriptional start site
